# Genetic variation modifies risk for neurodegeneration based on biomarker status

**DOI:** 10.3389/fnagi.2014.00183

**Published:** 2014-08-04

**Authors:** Timothy J. Hohman, Mary Ellen I. Koran, Tricia A. Thornton-Wells, for the Alzheimer's Neuroimaging Initiative

**Affiliations:** Department of Molecular Physiology and Biophysics, Center for Human Genetics and Research, Vanderbilt University School of MedicineNashville, TN, USA

**Keywords:** Alzheimer's disease (AD), MRI, CSF biomarkers, gene-environment interaction, genomics, amyloid, tau proteins

## Abstract

**Background**: While a great deal of work has gone into understanding the relationship between Cerebrospinal fluid (CSF) biomarkers, brain atrophy, and disease progression, less work has attempted to investigate how genetic variation modifies these relationships. The goal of this study was two-fold. First, we sought to identify high-risk vs. low-risk individuals based on their CSF tau and Aβ load and characterize these individuals with regard to brain atrophy in an AD-relevant region of interest. Next, we sought to identify genetic variants that modified the relationship between biomarker classification and neurodegeneration.

**Methods**: Participants were categorized based on established cut-points for biomarker positivity. Mixed model regression was used to quantify longitudinal change in the left inferior lateral ventricle. Interaction analyses between single nucleotide polymorphisms (SNPs) and biomarker group status were performed using a genome wide association study (GWAS) approach. Correction for multiple comparisons was performed using the Bonferroni procedure.

**Results**: One intergenic SNP (rs4866650) and one SNP within the *SPTLC1* gene (rs7849530) modified the association between amyloid positivity and neurodegeneration. A transcript variant of *WDR11-AS1* gene (rs12261764) modified the association between tau positivity and neurodegeneration. These effects were consistent across the two sub-datasets and explained approximately 3% of variance in ventricular dilation. One additional SNP (rs6887649) modified the association between amyloid positivity and baseline ventricular volume, but was not observed consistently across the sub-datasets.

**Conclusions**: Genetic variation modifies the association between AD biomarkers and neurodegeneration. Genes that regulate the molecular response in the brain to oxidative stress may be particularly relevant to neural vulnerability to the damaging effects of amyloid-β.

## Introduction

Competing models of the sporadic Alzheimer's disease (AD) cascade have debated whether the two primary pathologies, amyloid-beta (Aβ) plaques and tau neurofibrillary tangles, are causally related, with some suggesting that early amyloid pathology drives later tau pathology and others suggesting both pathologies arrive through distinct, unrelated molecular pathways (Small and Duff, 2008; Jack et al., 2010, 2013). In either case, it is theorized that the onset of these protein pathologies ultimately drives synaptic changes and the neurodegenerative cascade resulting in cognitive impairment.

Cerebrospinal fluid (CSF) measures of pathology *in vivo* have been applied alongside magnetic resonance imaging (MRI) to elucidate the relation between biomarkers of pathology and neurodegeneration and as combined measures of risk for disease onset and progression. To date, higher levels of CSF tau and lower levels of CSF Aβ have been shown to predict decreases in total and regional brain volume longitudinally, though regional patterns of atrophy appears to vary across diagnostic categories (Tosun et al., 2010). Other results have suggested that decreased CSF Aβ levels are related to atrophy rates in healthy normal adults (Fagan et al., 2009; Fjell et al., 2010; Schott et al., 2010), whereas either biomarker can predict atrophy in Mild Cognitive Impairment (MCI) and AD (Sluimer et al., 2010). Parallel research has demonstrated that the combined diagnostic utility of both CSF and MRI biomarkers is greater than either measure independently (Vemuri et al., 2009a,b; Sluimer et al., 2010; Davatzikos et al., 2011). Moreover, both CSF and MRI biomarkers appear to provide independent contributions to AD diagnosis, further suggesting each marks a distinct biological process in the AD cascade (Schoonenboom et al., 2008).

While a great deal of work has gone into understanding the relationship between CSF biomarkers, brain atrophy, and disease progression, few studies have attempted to investigate how genetic variation modifies these relationships. One possibility is that single genes are associated with biomarker load. Indeed, previous work has demonstrated a relationship between CSF Aβ and the *APOE* genotype (Morris et al., 2010), and more recently genome-wide association studies (GWAS) have identified genetic variants that are related to CSF biomarkers (Kim et al., 2011). A second possibility is that genes interact to confer risk or resilience from biomarker load. Work in our lab has identified novel gene-gene interactions that are related to Aβ load as measured with positron emission tomography (PET) (Hohman et al., 2013, 2014b; Koran et al., 2014b) and brain atrophy measured with MRI (Meda et al., 2013; Koran et al., 2014a). A final possibility is that genes interact with the presence of biomarkers to confer risk or resilience from longitudinal changes in brain volume or cognition. In one study, the *APOE* genotype in combination with biomarker positivity (high CSF tau or low CSF Aβ-42) at baseline was associated with increased regional atrophy in MCI subjects; however, the authors reported that no biomarker × *APOE* interaction reached statistical significance (Tosun et al., 2010). We previously identified an interaction between CSF levels of phosphorylated tau (ptau) and variation in the protection of telomeres 1 (*POT1*) gene that modified the association between ptau load and neurodegeneration (Hohman et al., 2014a). Yet, no study to date has systematically approached genetic modification of the relationship between CSF biomarkers of protein pathology and MRI biomarkers of disease progression.

The goal of this study was to identify genetic variants that modified the relationship between biomarker classification and neurodegeneration. We hypothesized we would identify a subset of individuals who were resilient to the neurodegenerative cascade associated with biomarker positivity based on genotypic variation across the sample. Moreover, we hypothesized that such variation would explain variance in brain atrophy above and beyond the effect of the *APOE* genotype. The identification of such genetic factors could clarify the mechanistic relationship between CSF biomarkers and neurodegeneration and provide targets for clinical intervention aimed at altering such pathways of neuro-vulnerability.

## Materials and methods

Data used in the preparation of this article were obtained from the Alzheimer's Disease Neuroimaging Initiative (ADNI) database (adni.loni.usc.edu). The ADNI was launched in 2003 by the National Institute on Aging (NIA), the National Institute of Biomedical Imaging and Bioengineering (NIBIB), the Food and Drug Administration (FDA), private pharmaceutical companies and non-profit organizations, as a $60 million, 5-years public-private partnership. The primary goal of ADNI has been to test whether serial MRI, PET, other biological markers, and clinical and neuropsychological assessment can be combined to measure the progression of MCI and early AD. Determination of sensitive and specific markers of very early AD progression is intended to aid researchers and clinicians to develop new treatments and monitor their effectiveness, as well as lessen the time and cost of clinical trials.

The Principal Investigator of this initiative is Michael W. Weiner, MD, VA Medical Center and University of California – San Francisco. ADNI is the result of efforts of many co-investigators from a broad range of academic institutions and private corporations, and subjects have been recruited from over 50 sites across the U.S. and Canada. The initial goal of ADNI was to recruit 800 subjects, but ADNI has been followed by ADNI-GO and ADNI-2. To date these three protocols have recruited over 1500 adults, ages 55–90, to participate in the research, consisting of cognitively normal older individuals, people with early or late MCI, and people with early AD. The follow up duration of each group is specified in the protocols for ADNI-1, ADNI-2 and ADNI-GO. Subjects originally recruited for ADNI-1 and ADNI-GO had the option to be followed in ADNI-2. For up-to-date information, see www.adni-info.org.

### Subjects

Participants were enrolled based on criteria outlined in the ADNI protocol (http://www.adni-info.org/scientists/ADNIStudyProcedures.aspx). To avoid spurious genetic effects due to population stratification, only Caucasian participants were used in analyses. Demographic data are presented in Table 1. All analyses were performed in the combined dataset which includes participants in both the ADNI-1 and ADNI-2/GO protocols. ADNI and ADNI-2/GO were approved by the Institutional Review Boards of all of the participating institutions. Informed written consent was obtained from all participants at each site. All data analyzed herein were de-identified and all analyses were deemed exempt by the Vanderbilt IRB per 45 CFR 46.101(b).

**Table 1:**
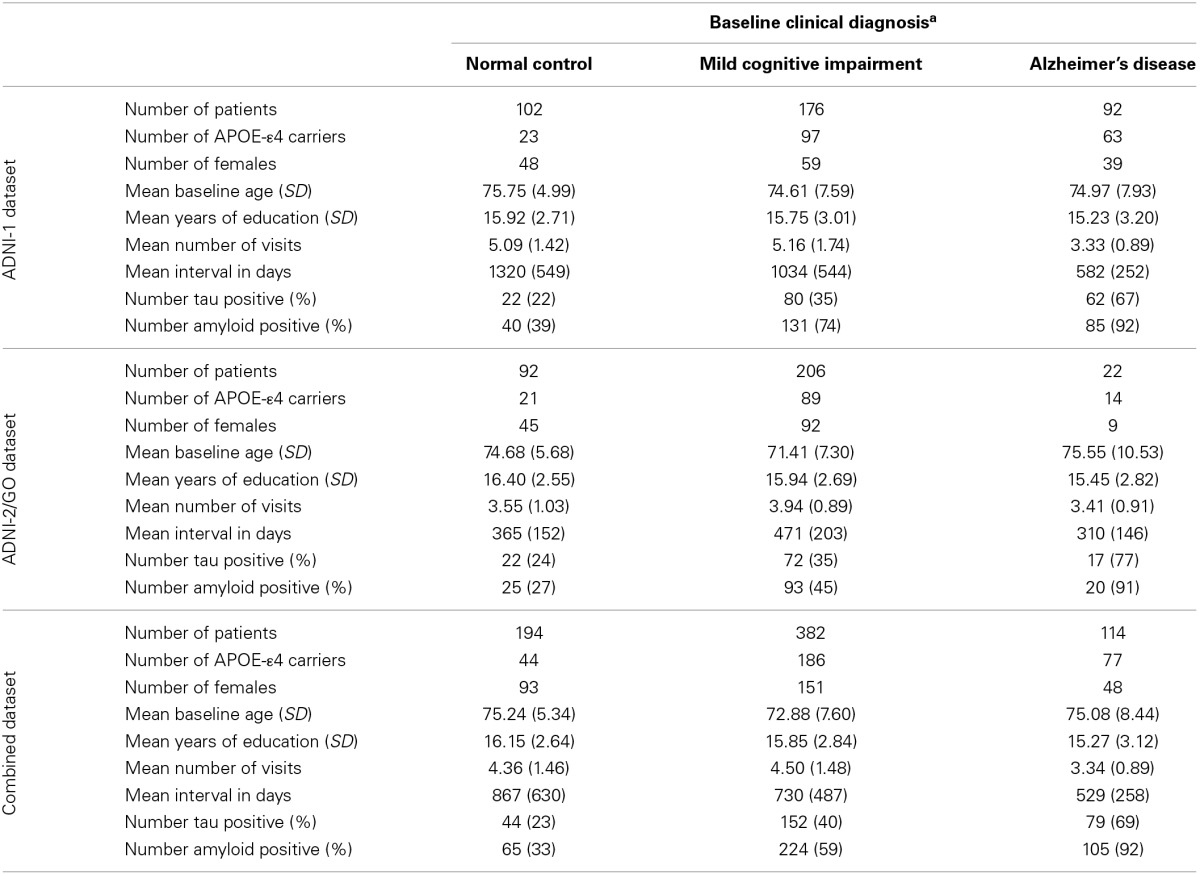
**Sample characteristics**.

a *Normal Control subjects had a Mini-Mental Status Examination (MMSE) score between 24 and 30, a Clinical Dementia Rating (CDR) score of 0, and were not depressed (Geriatric Depression Scale score < 6). Mild Cognitive Impairment subjects had a MMSE score between 24 and 30, objective memory impairment, subjective memory impairment, and a CDR score of 0.5. Alzheimer's Disease subjects met clinical criteria for dementia, had an MMSE of between 20 and 26, and had CDR score of 0.5 or 1*.

### Genotyping

In ADNI-1, genotyping was performed using the Illumina Infinium Human-610-Quad BeadChip. In ADNI-2/GO, genotyping was performed on the Illumina OmniQuad array. Quality control (QC) and statistical analyses were performed using PLINK software (version 1.07; Purcell et al., 2007). During QC, we excluded Single Nucleotide Polymorphisms (SNPs) with a genotyping efficiency <90%, a minor allele frequency (MAF) < 5%, or deviation from Hardy-Weinberg Equilibrium (*p* < 1 × 10^−6^). This left 515,383 SNPs in ADNI-1 and 605,317 SNPs in ADNI-2/GO. Finally, we merged the two genotyping files for our analyses and again applied a genotyping efficiency <90%. This left a total of 296,267 SNPs for data analysis.

### Quantification of ventricular dilation

We used all FreeSurfer data from 1.5 Tesla scans across ADNI-1, ADNI-GO, and ADNI-2 in our analyses by merging the publicly available FreeSurfer data for the two cohorts. Cortical reconstruction and volumetric segmentation were performed with the FreeSurfer image analysis suite version 4.3 (http://surfer.nmr.mgh.harvard.edu/; Dale et al., 1999; Fischl et al., 1999a,b). FreeSurfer processing in ADNI has been described in detail elsewhere (Mormino et al., 2009). An early version of the longitudinal image processing framework was used to process the sequential scans (Reuter et al., 2012). We used the change in volume of the left inferior lateral ventricle as our primary outcome measurement and included a measurement of intracranial volume (ICV) as a covariate in all volumetric analyses; both of which were defined in FreeSurfer (Desikan et al., 2006). Slopes of change in left ventricular volume over time were calculated in SAS 9.3 (SAS Institute Inc., Cary, NC) using mixed model regression (PROC MIXED) to leverage the longitudinal data available. A conventional mixed model was used that included the fixed effect of time (fraction of years from baseline) and the intercept, as well as a random effect for time and the intercept. In such a model, the assumption is that individual slopes are normally distributed with the fixed effect of time representing the mean, and the variance represented in the random effect. On average we had four MRI scans for each subject. More details on the longitudinal data are presented in Table 1.

### Biomarker groups

CSF biomarker quantification in ADNI was performed previously (Shaw et al., 2011), and detailed processing steps are available elsewhere (Jagust et al., 2009). The present dataset was compiled across UPENN1—UPENN5 data sources available for download from the ADNI site. We made use of the first available measure of Aβ-42 and total tau for each subject. The first observation for all ADNI-2/GO subjects came from the UPENN5 dataset; while the first observation for all ADNI-1 subjects came from the UPENN1 dataset. Three subjects in ADNI-1 did not have a useable observation in the UPENN1 dataset, so we used the first observation available from the other UPENN datasets. Subjects were classified into four groups based on previously defined cut-off points (Jagust et al., 2009): amyloid positive (Aβ − 42 ≤ 192), tau positive (tau ≥ 93), both amyloid and tau positive, or both amyloid and tau negative. We chose to use t-tau rather than p-tau in our biomarker groups because t-tau showed better sensitivity and specificity in the report establishing these cuts points (Jagust et al., 2009). Of the 690 participants analyzed, 234 were amyloid and tau negative, 160 were amyloid positive, 41 were tau positive, and 255 were both amyloid and tau positive.

### Statistical analyses: biomarker groups in relation to brain volume

Statistical analysis was performed using SPSS v. 22 (http://www-01.ibm.com/software/analytics/spss/). General linear model (GLM) was used to test the relation between biomarker groups and left inferior lateral ventricle volume (LILV). Past work has demonstrated that, when measured longitudinally, the LILV shows greater dilation than the right, both in AD patients and in controls (Thompson et al., 2004). We have also successfully applied this variable as a quantitative outcome in previous genetic interaction analyses (Hohman et al., 2014a; Koran et al., 2014a). LILV slopes were set as the quantitative outcome and biomarker group status was dummy coded where biomarker negative was set as the reference category. Sex, diagnosis, age, education, ICV, and *APOE* genotype (coded as carriers vs. non-carriers of the ε4 allele) were entered into the model as covariates.

### Statistical analyses: genetic interaction with biomarker group

Genetic interaction analyses were performed using the—linear command in PLINK. A dominant model was used for gene coding (0—no minor allele present, 1—minor allele present). The dominant model was selected to reduce the risk of spurious associations due to low contingency table cell counts when evaluating the biomarker group × SNP interaction. The same covariates were used for all genetic analyses; however, we excluded the *APOE* genotype at this stage in order to maximize our power to identify novel SNP effects. Biomarker groups were dummy coded with “biomarker negative” set as the reference category (LILV Slope = β_0_ + β_1_ Baseline_Age + β_2_ Baseline_ICV + β_3_ Gender + β_4_ Education + β_5_ Dx + β_6_ Tau_Positive + β_7_ Amyloid_Positive + β_8_ Tau_and_Amyloid_Positive + β_9_ SNP + β_10_ SNP^*^Tau_Positive + β_11_ SNP^*^Amyloid_Positive + β_12_ SNP^*^Tau_and_Amyloid_Positive). β_10−12_ were the terms of interest, and correction for multiple comparisons was performed for the total number of SNPs tested (296,109) using the Bonferroni procedure (cut-off *p*-value = 1.86 × 10^−7^). Next, the “Tau_and_Amyloid_Positive” term as removed from the model so that we could investigate SNPs that modify the relationship between these pathologies irrespective of the presence of the other pathology. In this case the amyloid term was coded as positive or negative, and the tau term was coded as positive or negative. Again, correction for multiple comparisons was performed using the Bonferroni procedure. Finally, these same two models were evaluated for SNP × biomarker interactions on baseline brain volume by setting baseline LILV as the quantitative outcome.

Although we do not have an independent replication sample with CSF data, MRI data, and genotype data, we chose to evaluate the consistency of our signal across datasets in order to provide some preliminary validation of our findings. The sample was divided into ADNI-1 and ADNI-2/GO based on the genotype chip used (Table 1), and significant interactions were re-evaluated within each cohort using the same covariates outlined above.

### *Post-hoc* analyses: hierarchical linear regression

Following genetic interaction analyses, hierarchical linear regression was performed in SPSS 22 in order to calculate the amount of variance explained by these novel genetic interactions above and beyond known predictors of brain volume and the *APOE* genotype. The first step in the model included sex, education, diagnosis, age, ICV, diagnosis, and biomarker group. Next, *APOE* genotype was inserted into the model. Third, the SNP term was inserted into the model. Finally, the SNP × Biomarker group term was added into the model. Change in R square was calculated at each step of the regression model.

## Results

### Biomarker groups in relation to brain volume

**Figure 1:**
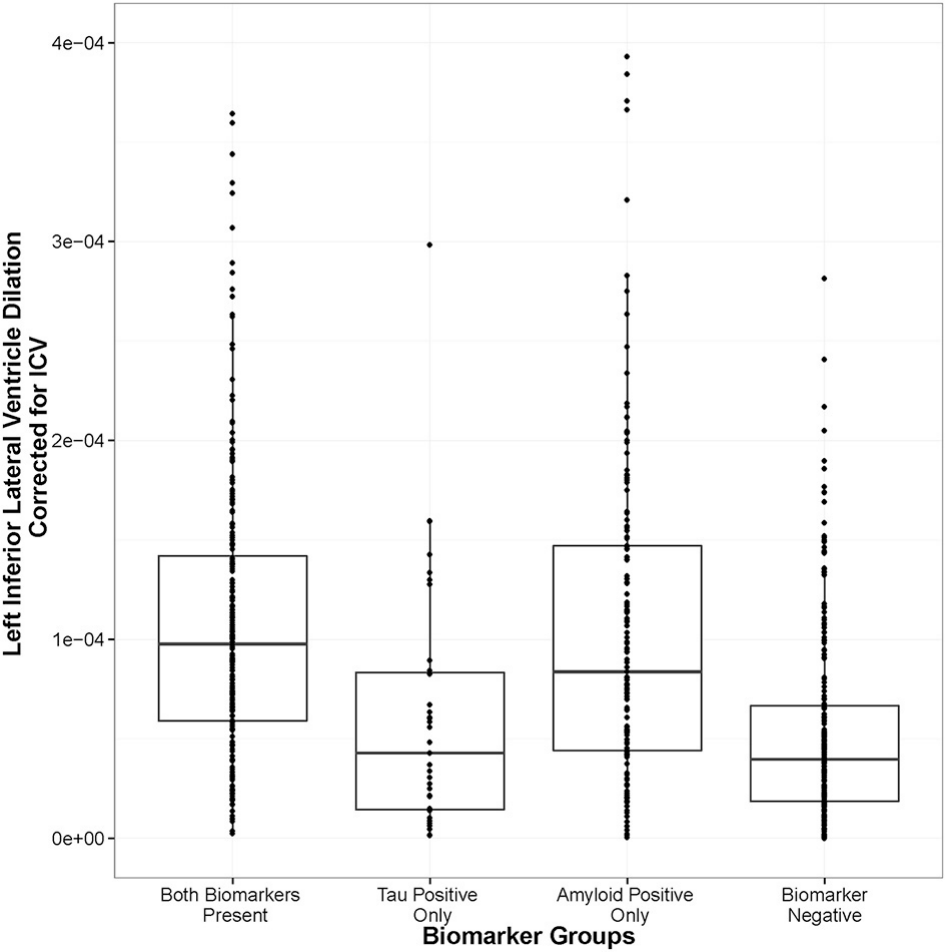
**Amyloid positivity and combined biomarker positivity predict ventricular dilation**. Biomarker groups are along the x-axis and annual change in left inferior lateral ventricle volume is on the y axis. The groups differ in their mean annual change, [*F*_(3, 679)_ = 6.50, *p* = 0.0002], with the amyloid positivity alone (*t* = 4.144, *p* = 0.00003) and the combined amyloid/tau positivity (*t* = 2.83, *p* = 0.005) groups showing significant deviation from the biomarker negative referent group.

As expected, biomarker group was associated with LILV slope when controlling for the covariates outlined above, [*F*_(3, 679)_ = 6.50, *p* = 0.0002]. As shown in Figure 1, amyloid positivity alone (*t* = 4.144, *p* = 0.00003) and combined amyloid/tau positivity (*t* = 2.83, *p* = 0.005) were associated with more rapid ventricular dilation relative to biomarker negativity, while tau positivity alone was not associated with a faster ventricular dilation (*t* = −0.22, *p* = 0.825). At baseline, biomarker group was also associated with LILV volume when controlling for the same variables [*F*_(3, 681)_ = 4.69, *p* = 0.003]. However, only amyloid positivity alone significantly differed from the biomarker negative group when controlling for multiple comparisons (*t* = 3.46, *p* = 0.003; Supplemental Figure 4). When modeling tau or amyloid positivity irrespective of the presence of the other biomarker (removing the “both” term from the model), only the amyloid term was associated with ventricular dilation (*t* = 4.38, *p* = 1 × 10^−5^).

### Genetic interaction analysis

**Figure 2:**
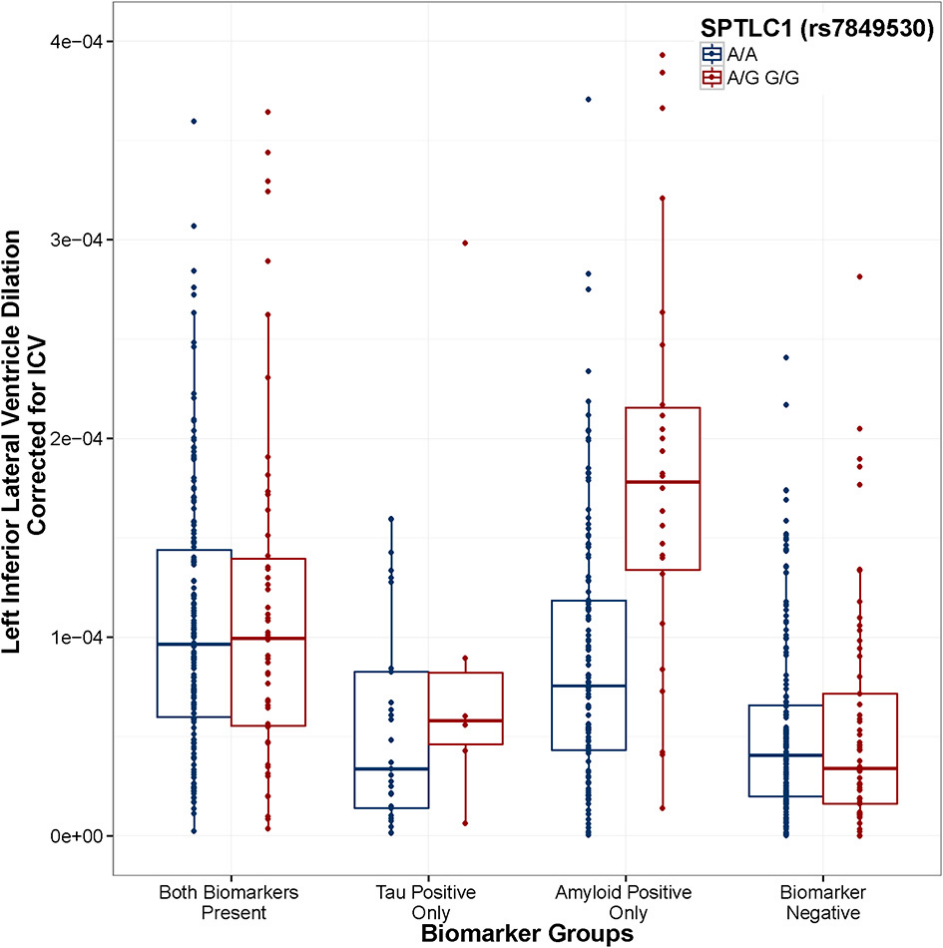
***SPTLC1* (rs7849530) modifies the association between amyloid positivity and ventricular dilation**. Biomarker groups are presented on the x-axis and annual change in the left inferior lateral ventricle is presented on the y-axis. Boxplots are grouped by rs7849530. G is the minor allele. When controlling for Age, Gender, Education, Diagnosis, and ICV, the amyloid_positive × rs7849530 interaction was statistically significant (*t* = 6.39, *p* = 3.14 × 10^−10^). In the amyloid-only biomarker group, carriers of the G allele showed a greater rate of ventricular dilation than homozygous carriers of the A allele (*t* = 5.306, *p* < 0.001). Model: LILV Slope = β0 + β1Baseline_Age + β2Baseline_ICV + β3Gender + β4Education + β5Dx + β6Tau_Positive + β7Amyloid_Positive + β8Tau_and_Amyloid_Positive + β9SNP + β10SNP^*^Tau_Positive + β11SNP^*^Tau_and_Amyloid_Positive + β12SNP^*^Amyloid_Positive.

**Figure 3:**
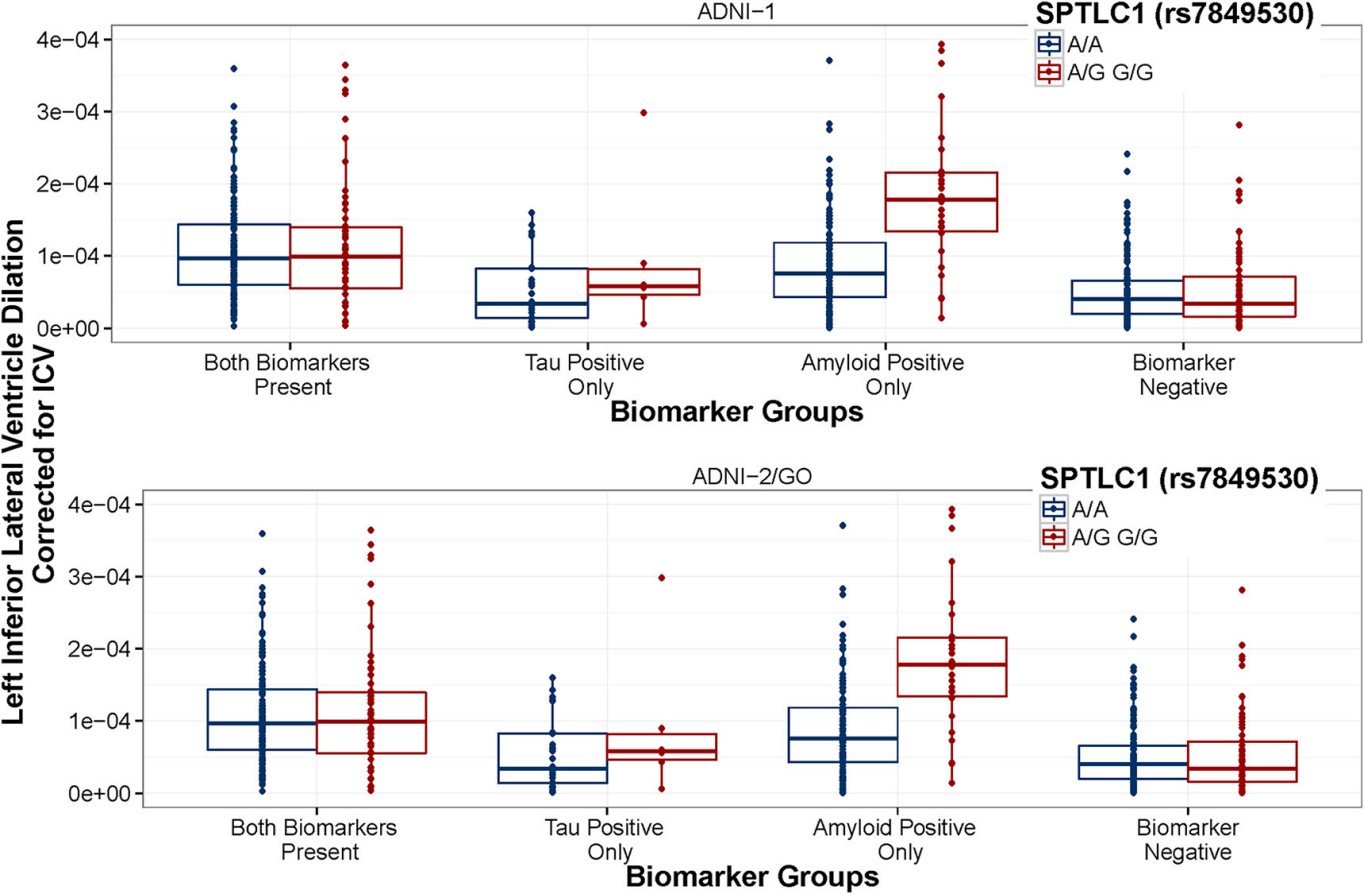
**SPTLC1 (rs7849530) interaction is consistent between datasets**. ADNI-1 data are presented in the top panel. ADNI-2/GO data are presented in the bottom panel. Biomarker groups are presented on the x-axis and annual change in the left inferior lateral ventricle is presented on the y-axis. Boxplots are grouped by rs7849530. G is the minor allele. The amyloid × rs7849530 interaction was statistically significant in both datasets (*p* < 0.01), and in both cases amyloid positive carriers of the G allele showed a greater rate of ventricular dilation than amyloid positive homozygous carriers of the A allele (*p* < 0.01).

When using the four group coding scheme (both positive, amyloid positive, tau positive, both negative), one SNP × Amyloid interaction showed an association with baseline ventricular volume: the intergenic SNP rs6887649 (MAF = 5%); however, this effect was not consistent across the ADNI-1 and ADNI-2/GO datasets (Table 2). Minor allele carriers showed larger ventricles at baseline in the amyloid positive group (Supplemental Figure 1). Two SNP × Amyloid interactions showed an association with longitudinal change in ventricular volume: the intergenic SNPs rs7849530 (MAF = 12%) and rs4866650 (MAF = 6%). Additional details of these results are presented in Table 2. These interactions remained statistically significant when correcting for MRI processing batch (*p* < 1.86 × 10^−7^). In both cases the presence of the minor allele was associated with faster dilation of the ventricles in amyloid positive individuals (Figures 2, 3).

**Table 2:** **SNP interaction results**.

**SNP**	**ADNI-1 dataset**		**ADNI2/GO dataset**		**Combined datasets**	
	***t***	***p*-value**	***t***	***p*-value**	***t***	***p*-value**
**AMYLOID INTERACTIONS ON INTERCEPT**						
rs6887649 (*FTMT*)	4.95	1.50 × 10^−6^	−0.13	0.899	5.68	1.99 × 10^−8^^*^
**AMYLOID INTERACTIONS ON SLOPE**						
rs7849530 (*SPTLC1*)	4.52	8.00 × 10^−6^	2.64	0.009	6.40	2.89 × 10^−10^^*^
rs4866650	3.89	0.0001	2.28	0.023	6.15	1.35 × 10^−9^^*^
**TAU INTERACTIONS ON SLOPE**						
rs12261764 (*WDR11-AS1*)	4.54	8.00 × 10^−6^	3.18	0.002	5.55	9.5 × 10^−8^^*^

* *Significant when correcting for multiple comparisons (Bonferroni < 0.05)*.

We also tested whether the peak observed interaction was clinically meaningful by testing whether it was associated with disease status as a binary outcome (rather than the quantitative outcome used in our original analyses). Indeed, the rs7849530 × amyloid interaction showed an association with disease status in a binary logistic regression with the same covariates used in the previous analyses (*OR* = 3.294, *p* = 0.046) suggesting that this interaction is also associated with clinical status.

**Figure 4:**
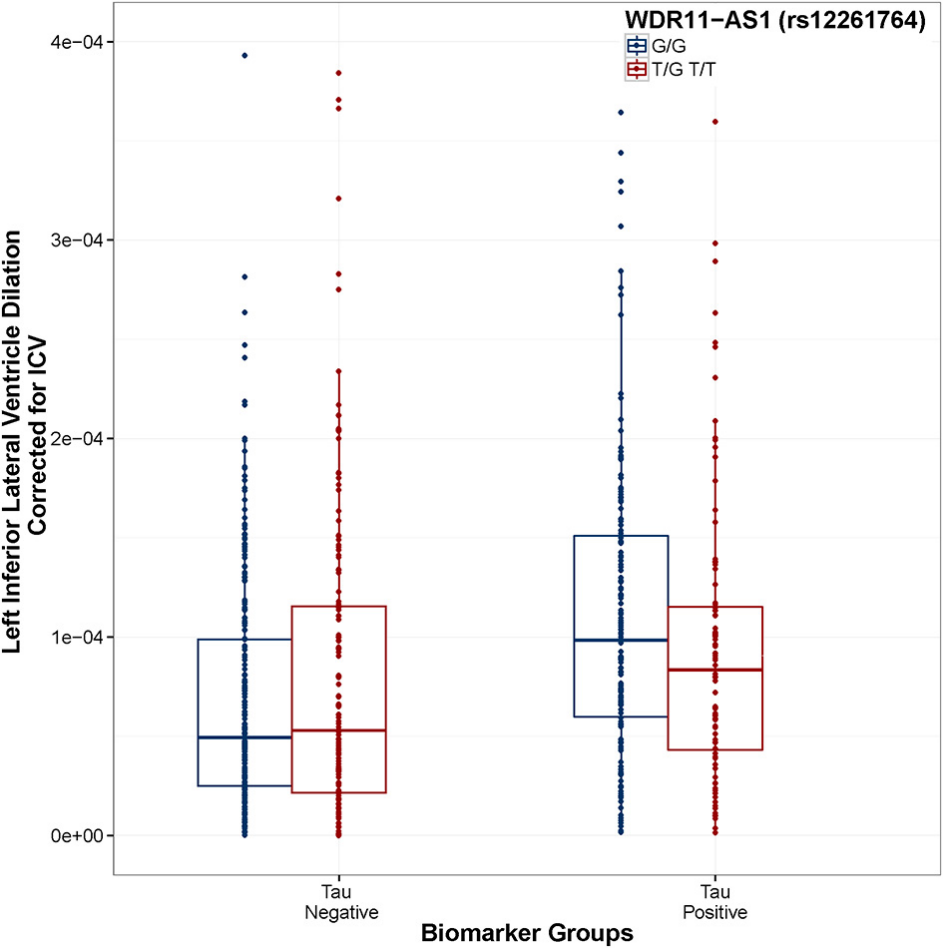
**WDR11-AS1 (rs12261764) modifies the association between Tau positivity and ventricular dilation**. Biomarker groups are presented on the x-axis and annual change in the left inferior lateral ventricle is presented on the y-axis. Boxplots are grouped by rs12261764. G is the minor allele. When controlling for Age, Gender, Education, Diagnosis, and ICV, the tau_positive × rs12261764 interaction was statistically significant (*t* = 5.55, *p* = 4.06 × 10^−8^). In the tau positive biomarker group, homozygous carriers of the G allele showed a greater rate of ventricular dilation than carriers of the T allele (*t* = 2.18, *p* = 0.030). In the tau negative biomarker group, homozygous carriers of the G allele showed a slower rate of ventricular dilation than carriers of the T allele (*t* = 2.21, *p* = 0.027). Model: LILV Slope = β0 + β1Baseline_Age + β2 Baseline_ICV + β3 Gender + β4 Education + β5Dx + β6Tau_Positive + β7Amyloid_Positive + β8SNP + β9SNP^*^Amyloid_Positive + β10SNP^*^Tau_Positive.

**Figure 5:**
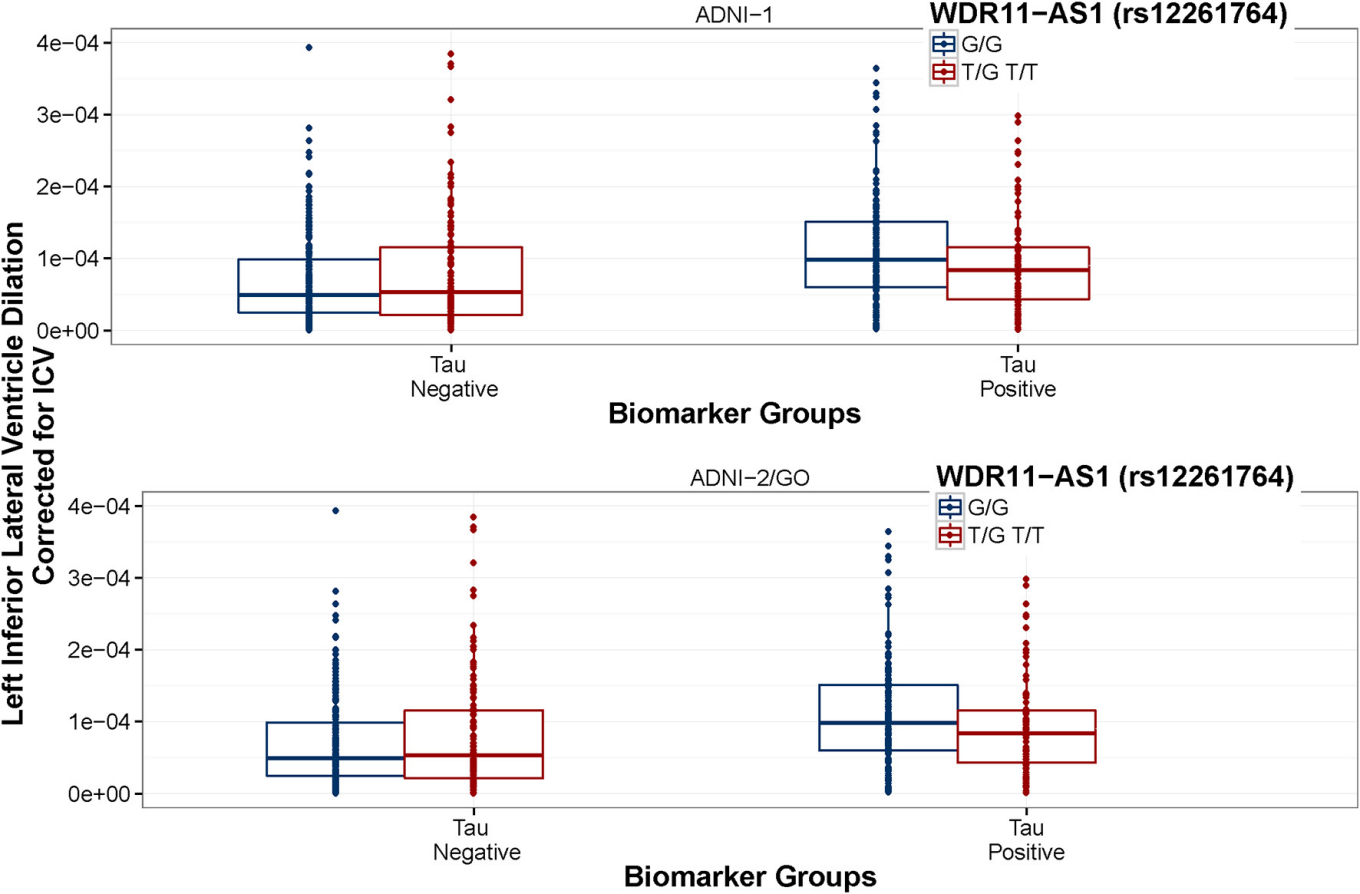
**WDR11-AS1 (rs12261764) interaction is consistent between datasets**. ADNI-1 data are presented in the top panel and ADNI-2/GO data are presented in the bottom panel. Biomarker groups are presented on the x-axis and annual change in the left inferior lateral ventricle is presented on the y-axis. Boxplots are grouped by rs12261764. G is the minor allele. The tau × rs7849530 interaction was statistically significant in both datasets (*p* ≤ 0.001). In ADNI-1, tau positive homozygous carriers of the G allele showed a greater rate of ventricular dilation than tau positive carriers of the T allele (*p* < 0.05), and the same trend was present in ADNI-2/GO (*p* = 0.059).

When using the second coding scheme (biomarker negative, tau positive, amyloid positive), no SNP × biomarker interactions were associated with baseline ventricular volume, however one SNP × Tau interaction showed an association with longitudinal change in ventricular volume: rs12261764 (MAF = 21%) annotated to *WDR11-AS1*. In this case, the minor allele was associated with slower dilation in tau positive individuals, and faster dilation in tau negative individuals (Figure 4), although only the difference in tau positive individuals showed consistency across the two ADNI subsets (Figure 5). This interaction also remained statistically significant when correcting for MRI processing batch (*p* < 1.86 × 10^−7^).

### *Post-hoc* analysis: hierarchical linear regression

Results are presented in Table 3. The first step in the hierarchical linear regression model explained 35.2% of the variance in LILV slope. In terms of the adjusted-R^2^, *APOE* genotype explained an additional 0.2% of variance. Rs7849530 explained an additional 1.9% of the variance, and the rs7849530 × biomarker group interaction terms explained an additional 3.5% of the variance. Similarly, rs4866650 explained 1.4% of variance and the rs4866650 × biomarker group interaction terms explained an additional 3.2% of variance. When using the second coding scheme (biomarker negative, tau positive, amyloid positive) the first step in linear regression model explained 35.7% of variance. *APOE* genotype explained 0.2% of variance. Rs12261764 explained less than 0.01% of variance alone, but the rs12261764 × biomarker interaction terms explained an additional 2.7% of variance.

**Table 3:** **Hierarchical linear regression results**.

**rs7849530**				**Change statistics**					
**Model**	**AIC**	***R*^2^**	**Adj. *R*^2^**	**Adj. *R*^2^ change**	**Adj. *R*^2^ change 95% CI^#^**	***F* change**	**df1**	**df2**	**Sig. *F C*hange (*P*-value)**
1^a^	8463	0.360	0.352	0.352	[0.30–0.39]	47.87	8	681	3.29 × 10^−61^
2^b^	8462	0.363	0.354	0.002	[−0.001–0.013]	2.83	1	680	0.092
3^c^	8442	0.382	0.373	0.019	[0.004–0.043]	21.83	1	679	4.00 × 10^−6^
4^d^	8444	0.384	0.373	0.000	[−0.001–0.005]	0.788	2	677	0.455
**5^e^**	**8406**	**0.419**	**0.408**	**0.035**	**[0.011–0.070]**	**40.798**	**1**	**676**	**3.14 × 10^−10^**

a *Predictors: Constant, Intracranial Volume, Age, Education, Diagnosis, Gender, Biomarker Group*.

b *Predictors: Constant, Intracranial Volume, Age, Education, Diagnosis, Gender, Biomarker Group, **APOE***.

c *Predictors: Constant, Intracranial Volume, Age, Education, Diagnosis, Gender, Biomarker Group, APOE, **rs7849530***.

d *Predictors: Constant, Intracranial Volume, Age, Education, Diagnosis, Gender, Biomarker Group, APOE, rs7849530, **rs7849530 × Tau_only, rs7849530 × Both***.

e *Predictors: Constant, Intracranial Volume, Age, Education, Diagnosis, Gender, Biomarker Group, eAPOE, rs7849530, rs7849530 × Tau_only, rs7849530 × Both, **rs7849530 × Amyloid_only***.

# *Ninety five percentage confidence interval calculated using a bootstrap procedure with 1000 replicates*.

## Discussion

Consistent with previous reports (Fjell et al., 2010), we found that amyloid positivity was a strong predictor of longitudinal change in ventricular volume, while tau positivity alone was not. Moreover, we have identified four SNPs that modify the association between biomarker positivity and neurodegeneration. These results suggest that genetic variation may alter individual susceptibility to the damaging effects of AD neuropathology, although future studies are needed to replicate the observed genetic interactions.

### Biomarker groups and longitudinal ventricular dilation

We observed an association between biomarker group and left ventricular dilation in which amyloid positivity alone or in combination with tau positivity was associated with longitudinal brain atrophy. This finding is consistent with previous reports, particularly in the inferior lateral ventricles, in which stronger associations have been observed between amyloid positivity and ventricular volume (Olt et al., 2010) and ventricular dilation (Fjell et al., 2010), while tau did not show this association. This observation is also consistent with the proposed cascade of AD biomarkers in which amyloid shows the earliest changes in biomarker levels, followed by tau (Jack et al., 2013). Thus, the observed association between the amyloid-only and the both biomarker group with brain volume change in an AD relevant region of interest is consistent with the expected AD cascade. The exact relation between CSF biomarkers and both cross-sectional and longitudinal brain volume appears to vary by brain region and is modified by genetic profile (Tosun et al., 2010). Thus, additional analyses targeting non-AD regions of interest, or performed at a voxel-wise level, may help further clarify the association between brain atrophy and biomarker status. Given the association also varies by diagnosis (Tosun et al., 2010), larger samples that allow stratification across diagnostic groups will be needed to fully evaluate potential genetic modifiers of this complex relationship. Regardless, the available data allowed us to pursue genetic modifiers of the association between biomarker status and brain volume and led to a few interesting interactions we will now discuss in detail.

### SNPs modify the association between amyloid positivity and ventricular volume

One SNP modified the association between amyloid positivity and ventricular volume at baseline (rs6887649). This intergenic SNP is 3 KB from a cluster of SNPs that have been associated with HDL-cholesterol levels in a recent GWAS (Kathiresan et al., 2007). This SNP is also 10 KB upstream of ferritin mitochondrial gene (*FTMT*). Interestingly, FtMt has been recently been implicated as a neuroprotective factor in neurodegenerative disease through regulation of iron homeostasis in the brain (Gao and Chang, 2014). Moreover, it has been specifically implicated in reducing oxidative damage and reducing β-amyloid-induced neurotoxicity (Wu et al., 2013). Our findings further implicate FtMt in AD pathogenesis, although a larger sample is needed to verify this effect, particularly given that the effect was not observed consistently between the ADNI-1 and ADNI-2/GO datasets.

Two SNPs were identified that modified the association between amyloid positivity and ventricular dilation (rs7849530 and rs4866650). In both cases these effects were consistent across the ADNI-1 and ADNI-2/GO cohorts, validating the association and suggesting the effect may indeed replicate in an independent sample. Interestingly, both of these SNPs are also within 50 kb of SNPs that have shown weak associations with AD previously (Li et al., 2008). Additionally, the rs7849530 × amyloid interaction showed an association with disease status in a binary logistic regression with the same covariates used in the previous model. As with brain volume, the minor allele at rs7849530 was protective in non-amyloid positive individuals and conferred risk in amyloid positive individuals.

Rs7849530 is 50 kb upstream of serine palmitoyltransferase, long chain base subunit 1 *(SPTLC1*). Interestingly, ceramide levels, both in the brain and the blood, have been associated with risk for AD in a number of studies (Satoi et al., 2005; Filippov et al., 2012; Mielke et al., 2012). Serine palmitoyltransferase is the rate limiting enzyme in ceramide synthesis, and *SPTLC1* has been specifically implicated in increased ceramide synthesis triggering apoptosis in Hereditary Sensory Neuropathy type 1 (HSN1) cells (Dawkins et al., 2001). Moreover, Aβ induced membrane oxidative stress has been shown to cause ceramide accumulation to increase, ultimately resulting in apoptosis and neurodegeneration (Cutler et al., 2004). The present results suggest that genetic variation associated with ceramide synthesis may leave individuals vulnerable to neurodegeneration in the presence of amyloid, perhaps due to the previously identified relation between Aβ induced oxidative stress and ceramide-induced apoptosis.

It is quite interesting that the higher rate of ventricular dilation in minor allele carriers of rs7849530 and rs4866650 is only observed in the group that is amyloid positive (only), but not in the group that is both amyloid and tau positive. In the current analyses we used baseline biomarker groups to predict future ventricular dilation, and past research has demonstrated that CSF biomarker changes in amyloid precede CSF biomarker changes in tau during the typical presentation of AD (Jack et al., 2013). Therefore, one possibility is that neural vulnerability due to ceramide levels may lead to an amyloid specific neurodegenerative process early in the disease process that is then compounded by a tau-specific neurodegenerative process later on. In such a scenario, a later starting point in an individual's disease course (when the paritcipant has both tau and amyloid pathology) could be associated with an increased rate of decline relative to non-risk groups (as in Figure 2), but may at that stage be a more tau-based process that would not be as impacted by a genetic predisposition toward higher levels of ceramides. Additional work following the course of neurodegenerative processes in relation to the course of CSF biomarker changes will be necessary to better understand how individual variability in genetic risk modifies this complex association.

### SNP modification of the relation between tau and neurodegeneration

The primary SNP associated with neurodegeneration in individuals with tau pathology was rs12261764 annotated to WDR11 antisense RNA 1 (*WDR11-AS1)*. In the hapmap sample, this SNP is in strong LD with rs122527599 (*R*^2^ = 0.98, *D*′ = 1), which has been shown to be an enhancer in fetal brain tissue (Ward and Kellis, 2012). WDR11 has been shown to interact with the transcription factor EMX1 leading to impaired development of olfactory neurons within individuals with Kallmann syndrome (Kim et al., 2010). There has been some indication of the presence of both tau and amyloid protein deposits in the olfactory bulb of individuals with AD (Mundiñano et al., 2011). It is unclear how such pathology might relate to the neurodegenerative cascade, or how the observed genetic interaction may modify such an association, but it provides an interesting target for future functional analyses.

### Strengths and weaknesses

The present manuscript provides evidence of multiple novel SNP × biomarker interactions in conferring risk or resilience from neurodegeneration. The joint analysis of the combined dataset allowed us to maximize our power (Skol et al., 2006), and the stratified *post-hoc* analyses provided support for the consistency of the observed effects across data sources. However, this manuscript is not without limitations. Although consistent effects were observed, a true replication sample from an external data source with GWAS and PET data will be necessary to confirm our findings. We did observe differences in effect size between ADNI-1 and ADNI-2/GO, likely due to the difference in follow-up time. It is also possible that differences in CSF batches between ADNI-1 and ADNI-2/GO could be driving the differences in effect size between the two groups. In the current analysis, it is difficult to distinguish batch effects from group differences because we used the first CSF observation for each subject (thus the batches align roughly with ADNI-1 subjects vs. ADNI-2/GO subjects). However, it should be noted that previous work has demonstrated the test-retest reliability of the biomarker measures from CSF in the ADNI dataset (Shaw et al., 2011). The proposed mechanisms also assume a strong association between CSF biomarker levels and levels of neuropathology (Clark et al., 2003; Strozyk et al., 2003). Future analyses will build on this finding by evaluating the observed interactions in an autopsy sample where a more direct relationship between genotype, gene expression, and AD pathology can be assessed.

In the present analysis we did not have a large enough sample to meaningfully assess gene-biomarker interactions across diagnostic groups. Our observed effects appeared to show similar trends across the diagnostic categories (Supplemental Figures 2, 3); however given the known differences in the relationship between brain volume and CSF biomarkers across diagnostic categories, it would be worthwhile to pursue gene-biomarker-diagnosis interactions, if a larger sample could be acquired.

An additional independent sample with a comparable longitudinal follow-up interval to that of ADNI-1 could help clarify whether the cohort differences observed are simply due to differences in power, or rather such differences were due to batch effects, MRI follow-up interval, or sample characteristics. Moreover, future work replicating our findings in an independent sample with MRI, CSF, and genotype data is needed to confirm the observed effects. Finally, to avoid possible confounding factors related to population substructure we chose to restrict all analyses to Caucasian individuals, and thus our results may not generalize to other ancestral populations. We believe the statistical approach taken provides a blue-print for future gene-environment interaction analyses aimed at identifying genetic modifiers of known AD risk factors.

### Author contributions

Timothy J. Hohman was responsible for design, analysis, interpretation, and drafting the manuscript. Mary Ellen I. Koran was responsible for design, interpretation, and revision of the manuscript. Tricia A. Thornton-Wells was responsible for design, interpretation, and revision of the manuscript.

### Conflict of interest statement

The authors declare that the research was conducted in the absence of any commercial or financial relationships that could be construed as a potential conflict of interest.
