# Supplementary material for: Genetic variation modifies risk for neurodegeneration based on biomarker status
Source: Front Aging Neurosci. 2014 Aug 4;6:183. doi: 10.3389/fnagi.2014.00183 (PMC4121544; doi:10.3389/fnagi.2014.00183)
Supplement: Supplementary file 1 [file Data_Sheet_1.DOCX]

## Supplemental Figure 1: FTMT (rs6887649) Modifies the Association between Amyloid Positivity and Ventricular Volume at Baseline.

**Supplemental Figure 1**: Biomarker groups are presented on the x-axis and baseline left inferior lateral ventricle volume is presented on the y-axis. Boxplots are grouped by *FTMT* genotype.


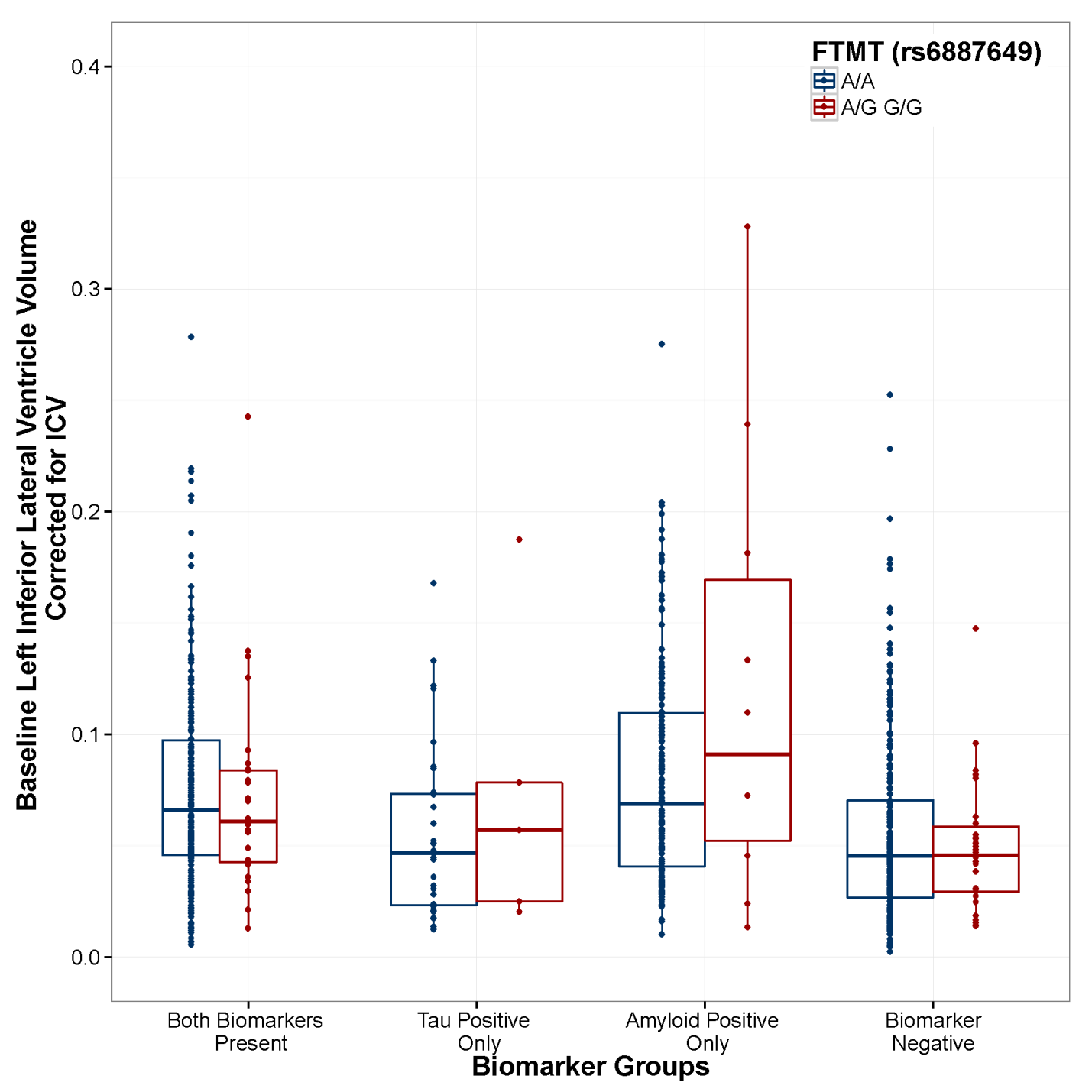


## Supplemental Figure 2: SPTLC1 (rs7849530) Interaction Stratified by Diagnosis.

**Supplemental Figure 2**: Biomarker groups are presented on the x-axis and annual change in the left inferior lateral ventricle is presented on the y-axis. Boxplots are grouped by *SPTLC1* genotype and are stratified by diagnostic category. Normal controls are on the top row, Mild Cognitive Impairment on the middle row, and Alzheimer’s Disease on the bottom row.


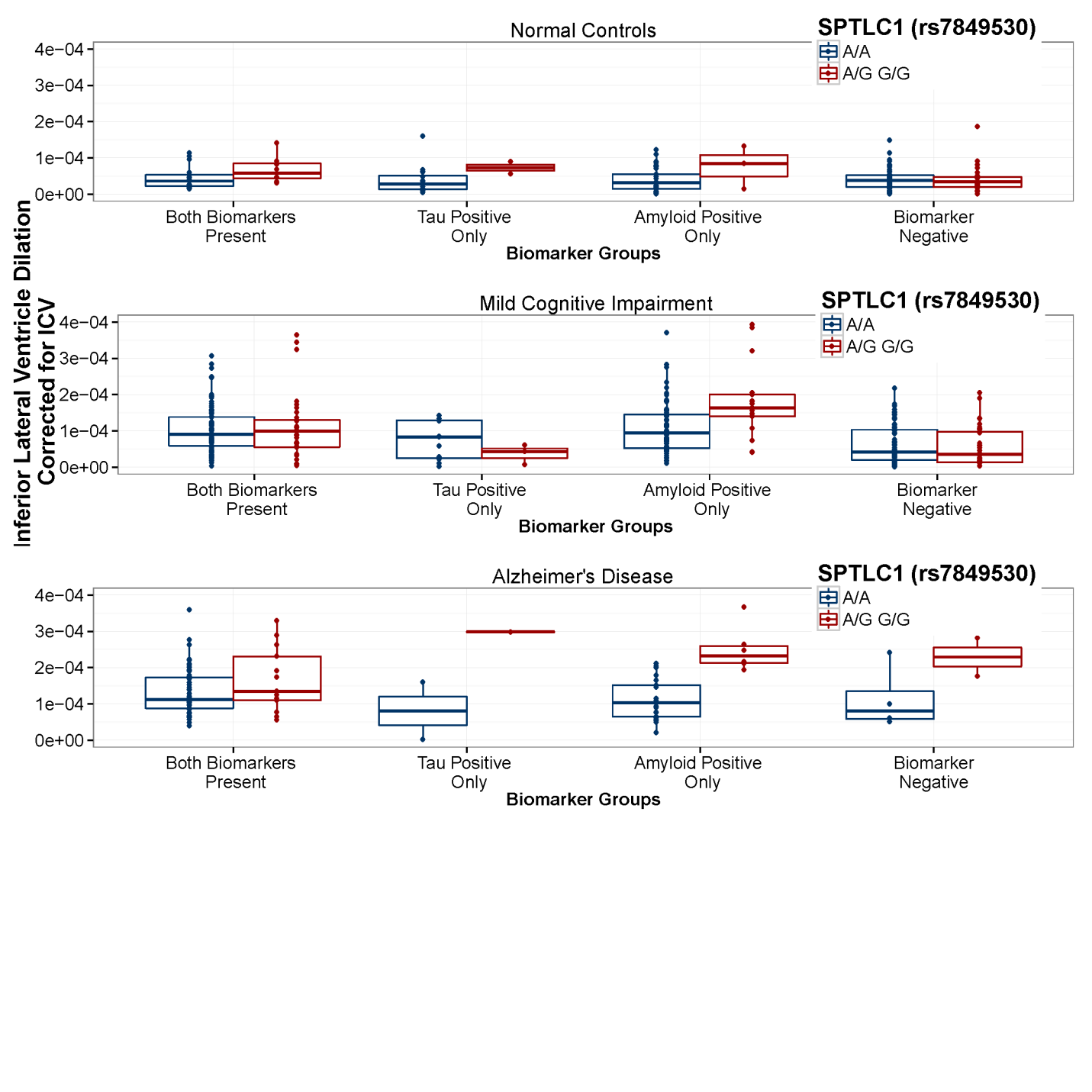


## Supplemental Figure 3: WDR11-AS1 (rs12261764) Interaction Stratified by Diagnosis.

**Supplemental Figure 3**: Biomarker groups are presented on the x-axis and annual change in the left inferior lateral ventricle is presented on the y-axis. Boxplots are grouped by *WDR11-AS1* genotype and are stratified by diagnostic category. Normal controls are on the top row, Mild Cognitive Impairment on the middle row, and Alzheimer’s Disease on the bottom row.


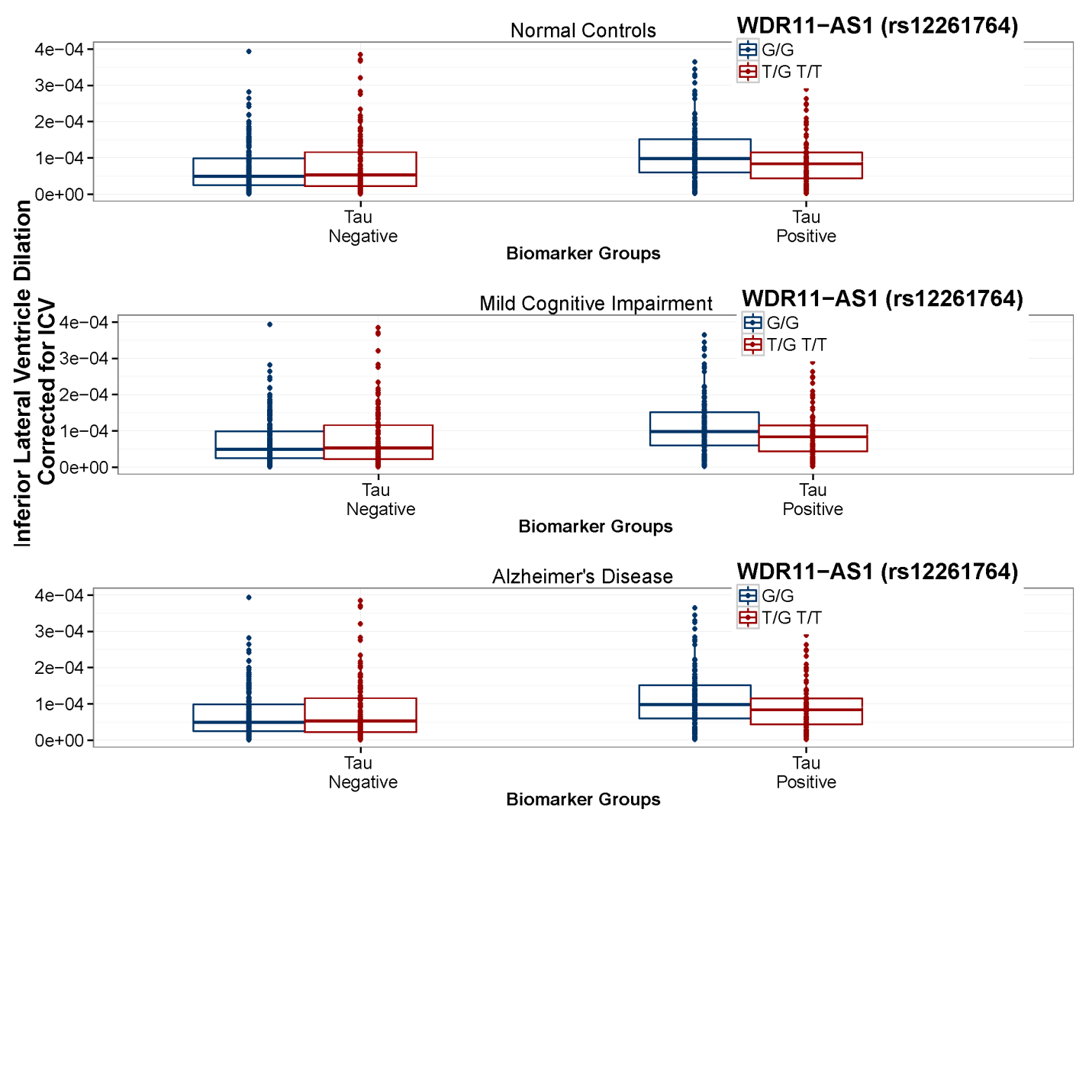


## Supplemental Figure 4: Amyloid Positivity and Combined Biomarker Positivity Predict Ventricular Volume at Baseline.

**Supplemental Figure 4**: Biomarker groups are along the x-axis and baseline left inferior lateral ventricle volume is on the y axis.
